# Endoscopic suture-based closure of a dehiscent rectal stump

**DOI:** 10.1055/a-2304-8401

**Published:** 2024-04-29

**Authors:** Alanna Ebigbo, Julia Wanzl, Shimaa Afify, Sandra Nagl, Helmut Messmann

**Affiliations:** 1 39694 Department of Gastroenterology, University Hospital Augsburg, Augsburg, Germany; 2 Department of Gastroenterology, National Hepatology and Tropical Medicine Research Institute, Cairo, Egypt

Lower anterior rectal resection (LAR) with discontinuity resection is performed to avoid leakage of the colorectal anastomosis in high risk patients 1 . Unfortunately, rectal stump leakage can occur, and reoperation is associated with high morbidity and mortality. Endoscopic techniques, such as rinsing or vacuum-based therapy, have shown promising results in rectal leak management 2 . Recently, various endoscopic suture-based techniques have been introduced to facilitate gastrointestinal defect closure and leak or fistula management 3 . We describe the case of a 66-year-old man with rectal stump leakage, presenting to our unit 12 days after LAR and discontinuity resection. We opted for a two-step approach with initial suturing of the large post-surgical defect using the through-the-scope X-Tack Endoscopic HeliX Tacking System (Apollo Endosurgery, Austin, Texas, USA) ( Video 1 ).

Endoscopic tack-and-suture technique for closure of a rectal stump dehiscence with a through-the-scope suturing device.Video 1

Endoscopy_UCTN_Code_TTT_1AQ_2AK

## Notes

Endoscopy E-Videos https://eref.thieme.de/e-videos
*E-Videos* is an open access online section of the journal *Endoscopy* , reporting on interesting cases and new techniques in gastroenterological endoscopy. All papers include a high-quality video and are published with a Creative Commons CC-BY license. Endoscopy E-Videos qualify for HINARI discounts and waivers and eligibility is automatically checked during the submission process. We grant 100% waivers to articles whose corresponding authors are based in Group A countries and 50% waivers to those who are based in Group B countries as classified by Research4Life (see: https://www.research4life.org/access/eligibility/ ). This section has its own submission website at https://mc.manuscriptcentral.com/e-videos .
